# Differential Prognostic Impact of Prior Cholecystectomy Between Proximal and Distal Colorectal Cancer: A 12‐Year Retrospective Cohort Study of 3487 Consecutive Patients

**DOI:** 10.1002/ags3.70190

**Published:** 2026-02-04

**Authors:** Masashi Tsunematsu, Koichiro Haruki, Kazuhiro Yanamoto, Takuo Takehana, Shunta Ishizaki, Mitsuru Yanagaki, Yoshihiro Shirai, Kenei Furukawa, Shinji Onda, Toru Ikegami

**Affiliations:** ^1^ Department of Digestive Surgery Saku Central Hospital Advanced Care Center Saku Nagano Japan; ^2^ Division of Hepatobiliary and Pancreatic Surgery, Department of Surgery The Jikei University School of Medicine Minato‐ku Tokyo Japan

**Keywords:** carcinogenesis, cholecystectomy, colorectal cancer, prognosis, tumor location

## Abstract

**Background:**

The prognostic impact of prior cholecystectomy (CCY) in patients with colorectal cancer (CRC) remains uncertain. The aim of this study was to evaluate the prognostic impact of prior CCY in a large cohort of patients with CRC, with particular attention to tumor location.

**Methods:**

We retrospectively analyzed all patients diagnosed with colorectal adenocarcinoma between January 2012 and December 2023. Patients were classified into proximal (cecum, ascending, and transverse) and distal (descending, sigmoid, and rectum) groups. Survival outcomes were compared using multivariate Cox proportional hazards regression models, including an interaction term between tumor location and history of CCY. Propensity score matching (PSM) was performed as a sensitivity analysis.

**Results:**

Among 3487 patients (1375 proximal and 2112 distal CRC), 158 (4.8%) had a history of CCY. Proximal CRC was more frequently associated with female sex, older age, history of CCY, poor differentiation, BRAF mutation, MSI‐high status, and non‐resection initial strategy (*p* ≤ 0.005). Among proximal CRC patients, multivariate analysis revealed TNM stage III/IV (*p* < 0.001), non‐resection initial strategy (*p* < 0.001), and history of CCY (*p* = 0.042) were independent predictors of poor overall survival (OS). Prior CCY was associated with worse OS in proximal CRC (*p =* 0.004). A significant interaction between tumor location and history of CCY was observed (*p* for interaction = 0.025).

**Conclusions:**

History of CCY was associated with worse prognosis in proximal CRC but not in distal CRC, suggesting a differential impact of CCY on prognosis in CRC. Our findings would provide insight into further research on bile acid metabolism or microbiome modulation in CRC patients.

## Graphical abstract

In this retrospective cohort of 3487 patients with CRC, history of CCY was associated with significantly worse prognosis in patients with proximal CRC but not in those with distal CRC. This differential prognostic impact showed a statistically significant interaction between tumor location and history of CCY and remained robust after PSM. Our findings would provide insight into future research on novel therapeutic interventions targeting bile acid metabolism or microbiome modulation specifically in high‐risk subgroup of proximal CRC with history of prior CCY.

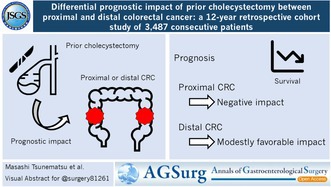

## 1 Introduction

Colorectal cancer (CRC) is one of the most common malignancies worldwide, and its prognosis is influenced by both tumor biology and host‐related factors [1, 2]. Cholecystectomy (CCY) is a common surgical procedure, and previous studies have suggested potential association between CCY and CRC carcinogenesis, particularly in the right‐sided (proximal) colon [3, 4, 5]. The underlying mechanism is thought to involve altered bile acid metabolism and changes in the gut microbiota, which may promote mucosal inflammation, DNA damage, and ultimately tumorigenesis [6, 7, 8].

CRC is a heterogeneous disease, and tumor location has emerged as an important determinant of prognosis and treatment response [9, 10]. Proximal CRC is more often associated with female sex, advanced age, microsatellite instability (MSI), and BRAF mutations, whereas left‐sided (distal) CRC is characterized by chromosomal instability and better responsiveness to certain systemic therapies [11, 12]. Given these biological and clinical differences, risk factors or exposures may exert differential prognostic effects depending on tumor location.

Although the association between a history of CCY and the risk of CRC development has been investigated, the prognostic impact of prior CCY in patients with CRC remains uncertain. Moreover, no large‐scale study has clarified whether this relationship differs according to tumor location. Recent evidence suggests that a history of CCY may be associated with increased tumor aggressiveness in proximal but not in distal CRC [13]. Therefore, we hypothesized that prior CCY exerts a location‐specific prognostic effect rather than a uniform influence across all CRCs. The present study aimed to evaluate the prognostic impact of prior CCY in a large cohort of patients with CRC, with particular attention to tumor location.

## 2 Methods

### 2.1 Patient Selection

All patients with colorectal adenocarcinoma diagnosed between January 2012 and December 2023 at Saku Central Hospital Advanced Care Center, the principal referral hospital for CRC in the eastern region of Nagano Prefecture, were included in this retrospective study. Patients were obtained from the institutional cancer registry, and duplicate cases were excluded. Patients were followed until death or the end of follow‐up. The study was approved by the Institutional Ethics Committee of the Saku Central Hospital Advanced Care Center [R202507‐03] and conformed to the provisions of the Declaration of Helsinki, as revised in Fortaleza, Brazil, October 2013. Informed consent was obtained from all participants.

### 2.2 Treatment Strategy for CRC

Initial treatment strategies, including resection or non‐resection approaches, were determined according to institutional practice and current Japanese clinical guidelines at the time of diagnosis. Specifically, decisions regarding endoscopic or surgical resection were based on a comprehensive assessment of tumor extent and anatomical resectability, patient performance status, comorbidities, and overall physiological reserve. All cases were discussed in a multidisciplinary conference involving gastroenterologists, surgeons, medical oncologists, and radiologists. Patients deemed unsuitable for upfront resection due to advanced disease, poor performance status, or severe comorbidities were managed with non‐resection strategies, including systemic therapy or best supportive care. Basically, RAS, BRAF, and MSI were assessed in patients with recurrence or metastases.

### 2.3 Data Acquisition

Data were collected from the clinical records of patients. The clinical variables extracted included age, sex, body mass index, history of diabetes mellitus, history of CCY, tumor location, tumor‐node‐metastasis (TNM) stage, histological differentiation, RAS and BRAF mutations, MSI, and initial treatment strategy. History of CCY was determined by reviewing the surgical records and confirming the absence of the gallbladder through abdominal computed tomography scans within the hospital portal or via imaging reports. Patients who underwent CCY within 2 years before the diagnosis of CRC were considered as having no history of CCY, taking into account the potential presence of occult CRC at the time of CCY [13]. Tumor location was categorized as proximal (cecum, ascending colon, and transverse colon) or distal (descending colon, sigmoid colon, and rectum) based on the previous studies [12, 13, 14]. Clinical stage was determined by combining each patient's TNM stages according to the 8th edition of the UICC classification. KRAS, NRAS, and BRAF mutation status were obtained from the molecular analysis reports of patients who presented with metastasis.

### 2.4 Statistical Analysis

Statistical analyses were performed using STATA/SE (version 14.2; StataCorp, College Station, TX, USA). We used the two‐sided α level of 0.05. Our primary analyses were assessment of the survival association of history of CCY with overall survival. All other test, including assessment of risk estimates, represented secondary analyses. Data are expressed as a median, interquartile range, or ratio. Continuous and categorical variables were compared using the Mann–Whitney U test, chi‐square test, or Fisher's exact test, as appropriate. Cumulative survival probabilities were estimated using the Kaplan–Meier method and compared using the log‐rank test. The cut‐off values for continuous variables were defined according to the guideline [14].

We assessed the association between overall survival (OS) and history of CCY in patients with CRC. Univariate and multivariate Cox regression analyses were used to estimate hazard ratios (HRs) for OS. The initial multivariate model included sex (female vs. male), age (≥ 65 vs. < 65 years), diabetes mellitus (yes vs. no), clinical TNM stage (III–IV vs. 0–II), tumor differentiation (poor vs. well/moderate), RAS mutation (yes vs. no), initial treatment strategy (resection vs. non‐resection), and history of CCY (yes vs. no). A backward elimination was conducted with a threshold *p* of 0.05 to select variables for the final models.

Next, the statistical interaction between tumor location (proximal vs. distal) and history of CCY (yes vs. no) was evaluated using the Wald test for the cross‐product in the multivariate‐adjusted Cox proportional regression model for overall survival.

Finally, PSM was performed as a sensitivity analysis to reduce bias inherent in this retrospective study. The propensity score was calculated using baseline characteristics in the logistic regression model. Baseline characteristics included age, sex, history of diabetes mellitus, clinical TNM stage, tumor differentiation, RAS mutation status, and initial treatment strategy. Patients with and without history of CCY were matched in a 1:2 ratio using a caliper of 0.100433.

## 3 Results

### 3.1 Patient Characteristics and Comparison of the Clinicopathological Variables According to Tumor Location

**FIGURE 1:**
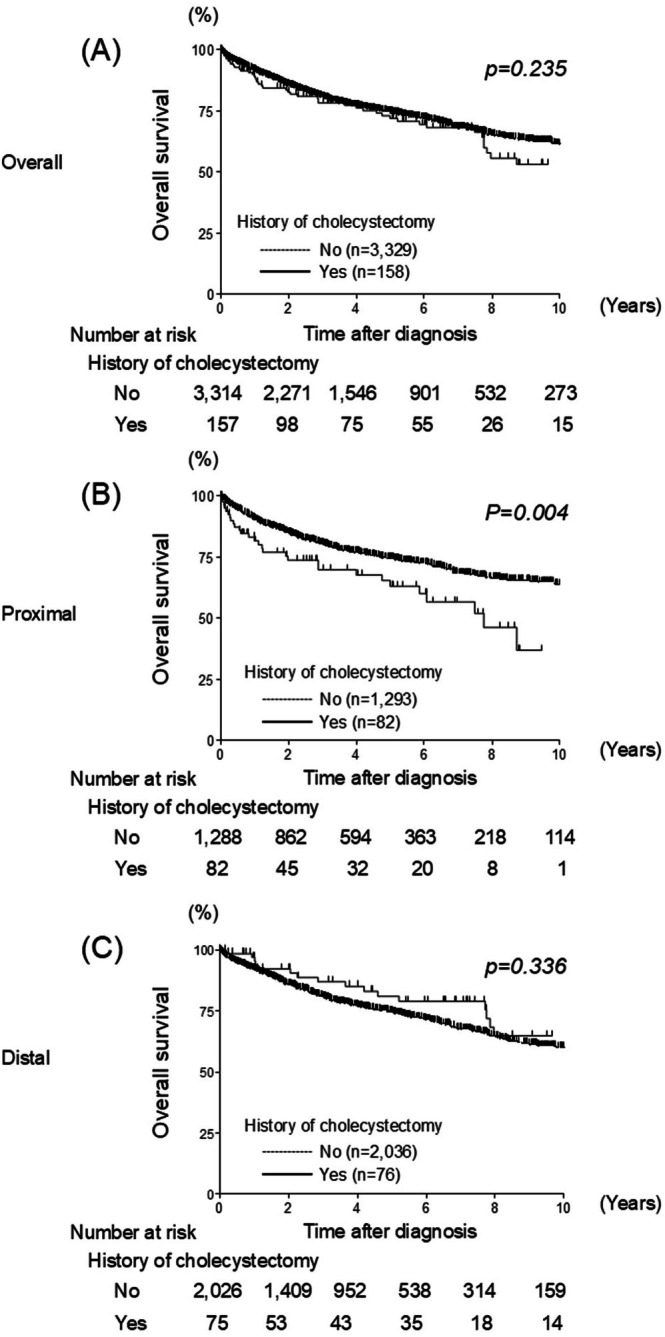
Kaplan–Meier curves of overall survival according to tumor location before propensity score matching. In proximal colorectal cancer (B), patients with a history of cholecystectomy had a significantly worse prognosis than those without (*p* = 0.004). By contrast, in the overall cohort (A) and in distal colorectal cancer (C), no significant difference in prognosis was observed between patients with and without cholecystectomy (*p* = 0.235 and *p* = 0.336, respectively).

Patient characteristics are outlined in Table 1. A total of 3487 patients were included: 1375 with proximal CRC and 2112 with distal CRC. Among all patients, 158 patients (4.5%) had history of CCY. Proximal CRC was more frequently associated with female (*p* < 0.001), older age (*p* < 0.001), history of CCY (*p* = 0.001), poor differentiation (*p* < 0.001), BRAF mutation (*p* < 0.001), MSI‐high status (*p* < 0.001), and non‐resection strategy (*p* < 0.001) compared with distal CRC. No significant difference in OS was observed according to CCY history in the entire cohort (*p* = 0.235) and in patients with distal CRC (*p* = 0.336). However, among patients with proximal CRC, a history of CCY was associated with significantly worse OS (*p =* 0.004, Figure 1).

**TABLE 1:** Comparison of the clinicopathological variables according to tumor location (*n* = 3487).

Variables	Overall (*n* = 3487)	Proximal (*n* = 1375)	Distal (*n* = 2112)	*p*
Sex, female	1444 (41%)	663 (48%)	781 (37%)	< 0.001
Age at diagnosis, years	63 (71–79)	67 (74–81)	62 (70–77)	< 0.001
Body mass index, kg/m^2^	20 (23–25)	20 (22–25)	20 (23–25)	0.741
Diabetes mellitus, yes	649 (19%)	263 (19%)	386 (18%)	0.528
History of cholecystectomy, yes	158 (4.5%)	82 (6%)	76 (4%)	0.001
Tumor location		Cecum 276 (20%)	Descending colon 172 (8%)	
		Ascending colon 725 (53%)	Sigmoid colon 1081 (51%)	
		Transverse colon 374 (27%)	Rectum 859 (41%)	
Clinical TNM stage				0.176
0	866 (25%)	367 (27%)	499 (24%)	
I	680 (20%)	270 (20%)	410 (19%)	
IIA	267 (7.7%)	104 (7.6%)	163 (7.7%)	
IIB	60 (1.7%)	21 (1.5%)	39 (1.9%)	
IIC	24 (0.7%)	8 (0.6%)	16 (0.8%)	
IIIA	97 (2.8%)	32 (2.3%)	65 (3.1%)	
IIIB	498 (14%)	192 (14%)	306 (15%)	
IIIC	283 (8.1%)	122 (8.9%)	161 (7.6%)	
IVA	361 (10%)	129 (9.4%)	232 (11%)	
IVB	245 (7%)	88 (6.4%)	157 (7.4%)	
IVC	55 (1.6%)	27 (2%)	28 (1.3%)	
Tumor differentiation				< 0.001
Well	1962 (56%)	729 (53%)	1233 (58%)	
Moderate	869 (25%)	315 (23%)	554 (26%)	
Poorly	153 (4.4%)	93 (6.8%)	60 (2.8%)	
Undifferentiated	503 (14%)	238 (17%)	265 (13%)	
KRAS mutation, yes	529 (42%)	202 (45%)	327 (62%)	0.058
NRAS mutation, yes	38 (4.1%)	9 (2.9%)	29 (4.8%)	0.166
BRAF mutation, yes	39 (6.9%)	31 (16%)	8 (2.2%)	< 0.001
MSI, high	52 (9.2%)	45 (23%)	7 (1.9%)	< 0.001
Initial treatment strategy				< 0.001
Resection	3108 (89%)	1865 (88%)	1243 (90%)	
Chemotherapy	201 (5.8%)	148 (7%)	53 (3.9%)	
Palliative care	178 (5.1%)	99 (4.7%)	79 (5.8%)	

Abbreviations: MSI, microsatellite instability; TNM, tumor node metastasis.

### 3.2 Univariate and Multivariate Analyses of Risk Factors for OS According to Tumor Location

In the univariate analysis of proximal CRC, age ≥ 65 years (*p* < 0.001), clinical TNM stage III–IV (*p* < 0.001), poorly differentiated tumor (*p* = 0.001), RAS mutation (*p* = 0.043), non‐resection initial strategy (*p* < 0.001), and history of CCY (*p* = 0.005) were associated with worse OS. In the multivariate analysis, clinical TNM stage III–IV (*p* < 0.001), non‐resection initial strategy (*p* < 0.001), and history of CCY (*p* = 0.042) were significantly associated with worse OS (Table 2). By contrast, in distal CRC, history of CCY was not associated with OS in neither univariate nor multivariate analyses (Table S1).

**TABLE 2:** Univariate and multivariate analyses of clinicopathologic variables in relation to overall survival after diagnosis in patients with proximal colon cancer (*n* = 1375).

Variables	N	Univariate analysis		Multivariate analysis	
		HR (95% CI)	*p*	HR (95% CI)	*p* *
Sex, female	663	1.11 (0.89–1.37)	0.332	—	—
Age, ≥ 65 years	1093	1.94 (1.42–2.64)	< 0.001	—	—
Diabetes mellitus, yes	263	0.95 (0.73–1.24)	0.710	—	—
Clinical TNM stage, IIII–V	590	3.34 (2.66–4.18)	< 0.001	2.88 (1.80–4.62)	< 0.001
Tumor differentiation, poor	93	1.84 (1.29–2.64)	0.001	—	—
RAS mutation, yes	213	1.40 (1.01–1.93)	0.043	—	—
Initial treatment strategy, resection	1243	0.07 (0.05–0.09)	< 0.001	0.18 (0.12–0.26)	< 0.001
History of cholecystectomy, yes	82	1.74 (1.19–2.54)	0.005	1.85 (1.02–3.36)	0.042

Abbreviations: CI, confidence interval; HR, hazard ratio; TNM, tumor node metastasis.

* The multivariate Cox regression model initially included sex (female vs. male), age (≥ 65 vs. < 65 years), diabetes mellitus (yes vs. no), clinical TNM stage (III–IV vs. 0–II), tumor differentiation (poor vs. well/moderate), RAS mutation (yes vs. no), initial treatment strategy (resection vs. non‐resection), cholecystectomy history (yes vs. no). Backward elimination was conducted with a threshold p of 0.05 to select variables for the final models.

### 3.3 Statistical Interaction Between History of CCY and Tumor Location in Relation to Overall Survival

We examined the statistical interaction between history of CCY and tumor location in relation to OS. We observed a statistically significant interaction between history of CCY and tumor location in OS analysis in both univariate and multivariate models (Table 3, *p* for interaction = 0.025). History of CCY was associated with poor OS in patients with proximal CRC [multivariate‐adjusted HR, 2.11; 95% confidence interval (CI), 1.13–3.92], but not in patients with distal CRC (multivariate‐adjusted HR, 0.63; 95% CI, 0.30–1.36).

**TABLE 3:** Association of cholecystectomy history with overall survival stratified by tumor location.

	*N*	Univariate	Multivariate**
		HR (95% CI)	HR (95% CI)
Proximal colon			
No history of cholecystectomy	1293	1 (referent)	1 (referent)
History of cholecystectomy	82	1.74 (1.19–2.54)	2.11 (1.13–3.92)
Distal colon			
No history of cholecystectomy	2036	1 (referent)	1 (referent)
History of cholecystectomy	76	0.80 (0.50–1.26)	0.63 (0.30–1.36)
*p*_interaction_ *		0.011	0.025

Abbreviations: CI, confidence interval; HR, hazard ratio.

* *p*_interaction_ value was calculated using the Wald test for the cross‐product of the Tumor location (proximal vs. distal) and history of cholecystectomy (Yes vs. No).

** Adjusted for the same set of covariates in Table 3.

### 3.4 Survival Analysis After PSM

As a sensitivity analysis, we conducted PSM analysis to investigate the impact of a history of CCY on survival in proximal or distal CRC patients. After PSM, baseline characteristics were well balanced in both proximal (Table S2) and distal (Table S3) CRC. After PSM, a history of CCY was associated with worse survival in proximal CRC (*p* = 0.040, Figure S1A), whereas favorable survival in distal CRC (*p* = 0.048, Figure S1B).

Given that treatment strategy may affect the outcomes, we further examined the association of a history of CCY with OS among patients who underwent resection in both proximal and distal CRC as additional sensitivity analysis. Because of the limited number of patients who did not undergo resection, PSM was applied only to resected cases. After PSM, baseline characteristics were balanced in both proximal (Table S4) and distal (Table S5) CRC. Among the resected cases, a history of CCY was associated with worse survival in proximal CRC (*p* = 0.005, Figure S2A), whereas no such association was observed in distal CRC (*p* = 0.422, Figure S2B).

## 4 Discussion

In this retrospective cohort of 3487 patients with CRC, we demonstrated that history of CCY was independently associated with worse OS in proximal CRC but not in distal CRC. This differential prognostic impact showed a statistically significant interaction between tumor location and history of CCY and remained robust after PSM. These findings highlight biological difference in effect of CCY on prognosis between proximal and distal CRC.

CCY fundamentally disrupts normal bile acid homeostasis by eliminating the gallbladder's storage and concentration functions, leading to continuous, unregulated bile flow directly into the duodenum [15]. This anatomical alteration results in sustained exposure of the proximal colon to secondary bile acids, such as deoxycholic acid, which exert their carcinogenic effects including DNA damage, oxidative stress, and tumor progression [3, 6, 7, 8, 12, 13]. In addition, bile acids activate key oncogenic signaling pathways, including Wnt/β‐catenin pathway [16], NF‐κB signaling [17], and engage bile acid receptors such as FXR and TGR5 [18]. These molecular interactions promote tumor growth, angiogenesis, and inflammatory responses that create a favorable environment for cancer progression. These direct tumorigenic effects are substantially amplified by profound alterations in the gut microbiome composition. Experimental and clinical studies have demonstrated that cholecystectomy induces long‐term alterations in gut microbiota composition, promoting bile‐tolerant and pro‐inflammatory bacterial species that enhance secondary bile acid production and colorectal carcinogenesis [4, 6, 7]. Importantly, accumulating evidence indicates that these bile acid–microbiome interactions are not only implicated in colorectal carcinogenesis, particularly in the proximal colon, but may also persist after tumor development and continue to shape a tumor‐promoting microenvironment that influences disease progression and prognosis. Elevated bile acid concentrations selectively promote the growth of pathogenic bacterial species capable of producing additional secondary bile acids, result in promoting inflammation. Concurrently, bile acid‐induced immune dysregulation manifests as suppression of antitumor lymphocyte activity and preferential recruitment of immunosuppressive cell populations, ultimately facilitating tumor immune evasion [19]. These biological mechanisms support our findings in which the proximal colon, anatomically positioned to receive the highest concentrations of bile acids following CCY, becomes particularly susceptible to developing a tumor‐promoting microenvironment that adversely impacts patient prognosis. Patients with proximal CRC and a history of CCY may therefore represent a clinically high‐risk subgroup that warrants particular attention in routine practice. Furthermore, these findings suggest that interventions targeting bile acid metabolism or the gut microbiota may have effects not limited to reducing the risk of colorectal carcinogenesis after cholecystectomy, but may also improve prognosis once proximal CRC has developed.

In contrast, the distal colon harbors a fundamentally different microbiome composition, characterized by an enrichment of beneficial butyrate‐producing bacterial species. These commensal organisms mediate potent anti‐inflammatory and antitumor effects through multiple protective mechanisms, including epigenetic regulation, apoptosis induction, and epithelial barrier reinforcement [20]. While bile acid alterations after CCY do reach the distal segments, the predominant butyrate‐producing microbiota can effectively counteract pro‐inflammatory signals and maintain homeostatic balance, thereby limiting the tumor‐promoting potential observed in the proximal colon. These location‐specific microenvironmental differences are further reinforced by distinct molecular and genetic landscapes between proximal and distal colorectal cancers. Recent evidence [19, 20] also suggests that gut microbiota may influence genomic instability, epigenetic regulation, and immune‐related molecular features in CRC, although direct causal links to specific oncogenic mutations remain to be fully elucidated. Proximal CRC characteristically exhibit MSI‐high status, BRAF mutations, and immune‐infiltrated but exhausted phenotypes that may be particularly susceptible to external carcinogenic stimuli. Conversely, distal CRC predominantly display chromosomal instability, EGFR pathway activation, and epithelial phenotypes that appear more resilient to bile acid‐induced perturbations [11, 12, 21, 22, 23, 24]. This comprehensive biological heterogeneity‐encompassing microbiome composition, immune landscapes, and molecular subtypes‐provides a mechanistic framework for understanding the differential vulnerability of proximal versus distal CRC to the adverse prognostic impact of CCY history observed in our study.

This study has several limitations. First, its retrospective single‐center design inherently limits external validity. Although treatment decisions were made according to clinical guidelines and based on comprehensive assessment of tumor extent, resectability, performance status, comorbidities, and multidisciplinary discussion, institutional practice patterns may still restrict the generalizability of our findings. Second, the potential for residual confounding bias cannot be entirely eliminated despite rigorous PSM. Unmeasured variables may have influenced the observed associations. In particular, key molecular characteristics such as BRAF, and MSI, were unavailable in a substantial proportion of patients and therefore could not be incorporated into the multivariable or PSM analyses, potentially affecting the robustness of the results. Third, the registry database did not fully capture detailed information on comorbidities, perioperative management, and adjuvant treatment, which may have affected survival outcomes. Forth, direct measurements of bile acid profiles and gut microbiome composition are lacking, which are central to the proposed biological mechanisms underlying our findings. In addition, the duration and magnitude of bile acid or microbiome exposure necessary to influence prognosis remain unclear. Although this two‐year cutoff was originally proposed to address carcinogenesis, we applied the same definition in prognostic analyses to minimize potential reverse causality and ensure that CCY preceded CRC development by a clinically meaningful interval. Fifth, this study was conducted in a homogeneous Japanese population, and the findings may not be directly generalizable to populations with different ethnic backgrounds. Previous studies in Asian cohorts have reported minimal association of CCY with CRC incidence [25, 26]. Although our study did not examine cancer development, the results that history of CCY was associated with prognosis in CRC patients provides an important clinical insight that warrants further validation in other populations. Finally, rectal cancer was classified into the distal CRC group in current study. While Japanese clinical guidelines often distinguish rectal cancer from colon cancer due to differences in surgical anatomy, operative techniques, and perioperative treatment strategies, we included rectal cancer in our analysis based on evidence from both our detailed subsite analysis and previous reports [21, 22] demonstrating that molecular characteristics change gradually along the colorectum, extending to the rectum. This approach is consistent with studies examining tumor sidedness in colorectal cancer [12, 13, 14].

Despite these limitations, this is, to our knowledge, one of the largest single‐institution analyses investigating the prognostic impact of history of CCY in CRC. Future studies should incorporate direct bile acid profiling, gut microbiome analysis, and diverse populations to validate these findings and elucidate the underlying biological mechanisms, ultimately translating these insights into personalized treatment approaches and improved clinical care for colorectal cancer patients.

## 5 Conclusion

In conclusion, history of CCY was associated with significantly worse prognosis in patients with proximal CRC but not in those with distal CRC, suggesting differential impact of CCY on prognosis in CRC patients. Although the biological mechanisms underlying the association between prior CCY and prognosis remain unclear, alterations in bile acid metabolism and the gut microbiota are plausible pathways.

Our findings would provide insight into future research on novel therapeutic interventions targeting bile acid metabolism or microbiome modulation specifically in high‐risk subgroup of proximal CRC with history of prior CCY.

## Author Contributions

**Masashi Tsunematsu:** conceptualization, data curation, investigation, writing – original draft, funding acquisition, formal analysis, project administration. **Koichiro Haruki:** conceptualization, investigation, writing – original draft, funding acquisition, formal analysis. **Kazuhiro Yanamoto:** data curation, writing – original draft. **Takuo Takehana:** writing – review and editing, data curation. **Shunta Ishizaki:** data curation, writing – review and editing. **Mitsuru Yanagaki:** data curation, writing – review and editing. **Yoshihiro Shirai:** data curation, writing – review and editing. **Kenei Furukawa:** conceptualization, writing – review and editing, methodology. **Shinji Onda:** investigation, writing – review and editing, methodology, supervision. **Toru Ikegami:** conceptualization, writing – review and editing, funding acquisition, supervision, methodology.

## Funding

This work was supported by JSPS KAKENHI Grants (JP22K16453 to M.T., 24K11920 to K.H., and 24K11898 to T.I.). The funders had no role in study design, data collection and analysis, decision to publish, or preparation of the manuscript.

## Ethics Statement

The study was approved by the Institutional Ethics Committee of the Saku Central Hospital Advanced Care Center [R202507‐03].

## Conflicts of Interest

The authors declare no conflicts of interest, except that Toru Ikegami serves as an editorial board member of the Annals of Gastroenterological Surgery.

## Supporting information


**Figure S1:** Kaplan–Meier curves of overall survival according to tumor location after propensity score matching including status of RAS mutation.
**Figure S2:** Kaplan–Meier curves of overall survival in patients who underwent resection according to the history of cholecystectomy.
**Table S1:** Univariate and multivariate analyses of clinicopathologic variables in relation to overall survival after diagnosis in patients with distal colon cancer (*n* = 2112).
**Table S2:** Comparison of the clinicopathological variables according to cholecystectomy history in patients with proximal colon cancer.
**Table S3:** Comparison of the clinicopathological variables according to cholecystectomy history in patients with distal colon cancer.
**Table S4:** Comparison of the clinicopathological variables according to cholecystectomy history in patients with proximal colon cancer who underwent resection.
**Table S5:** Comparison of the clinicopathological variables according to cholecystectomy history in patients with distal colon cancer who underwent resection.

## Data Availability

The data underlying this article cannot be shared publicly due to institutional policy and patient privacy concerns. The data are available from the corresponding author upon reasonable request and with permission of Saku Central Hospital Advanced Care Center.
