# Supplementary material for: Differential Prognostic Impact of Prior Cholecystectomy Between Proximal and Distal Colorectal Cancer: A 12‐Year Retrospective Cohort Study of 3487 Consecutive Patients
Source: Ann Gastroenterol Surg. 2026 Feb 4;10(4):1201–8. doi: 10.1002/ags3.70190 (PMC13327056; doi:10.1002/ags3.70190)
Supplement: Supplementary file 1 — Figure S1: Kaplan–Meier curves of overall survival according to tumor location after propensity score matching including status of RAS mutation. Figure S2: Kaplan–Meier curves of overall survival in patients who underwent resection according to the history of cholecystectomy. Table S1: Univariate and multivariate analyses of clinicopathologic variables in relation to overall survival after diagnosis in patients with distal colon cancer (n = 2112). Table S2: Comparison of the clinicopathological variables according to cholecystectomy history in patients with proximal colon cancer. Table S3: Comparison of the clinicopathological variables according to cholecystectomy history in patients with distal colon cancer. Table S4: Comparison of the clinicopathological variables according to cholecystectomy history in patients with proximal colon cancer who underwent resection. Table S5: Comparison of the clinicopathological variables according to cholecystectomy history in patients with distal colon cancer who underwent resection. [file AGS3-10-1201-s001.docx]

**Supplementary figure 1**

**
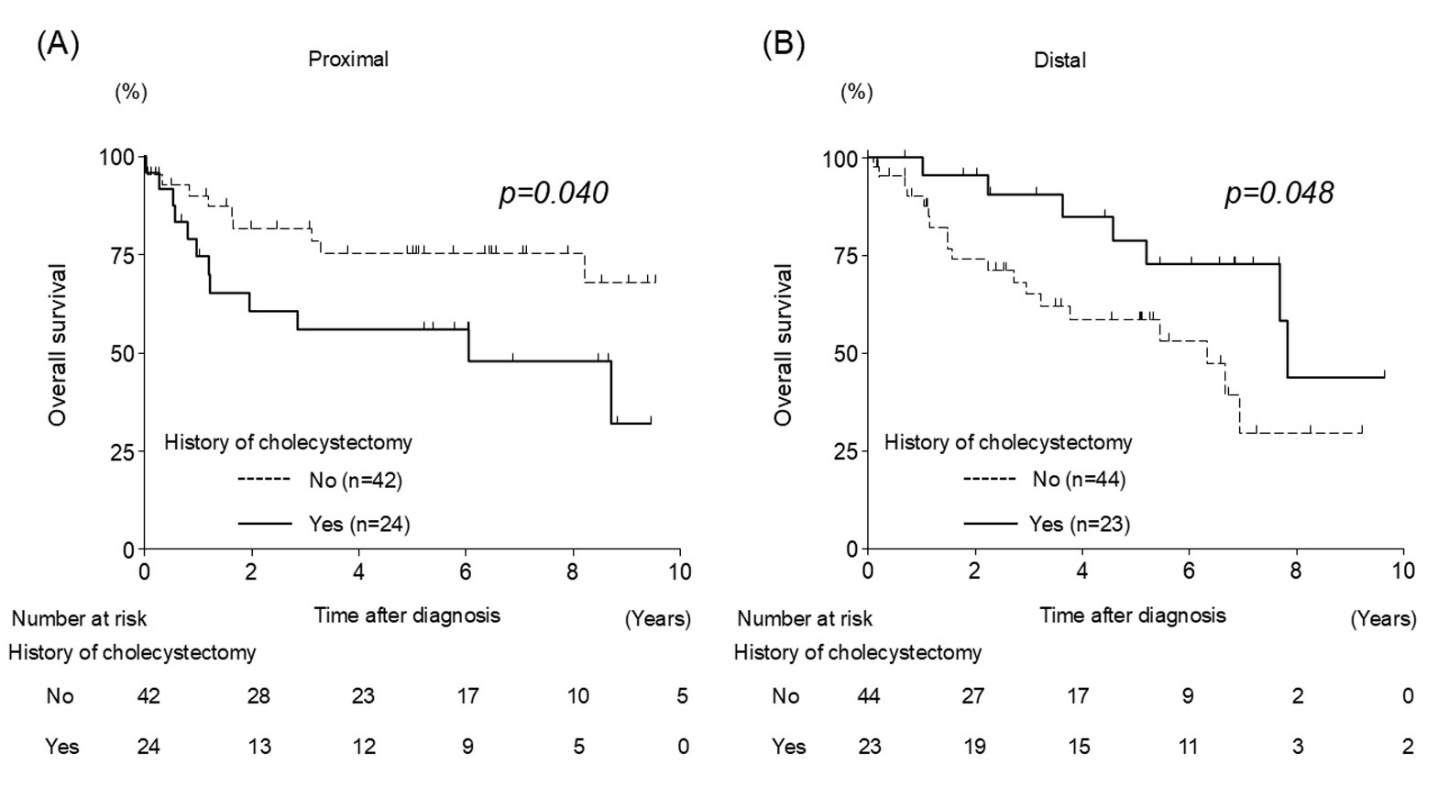
**

**
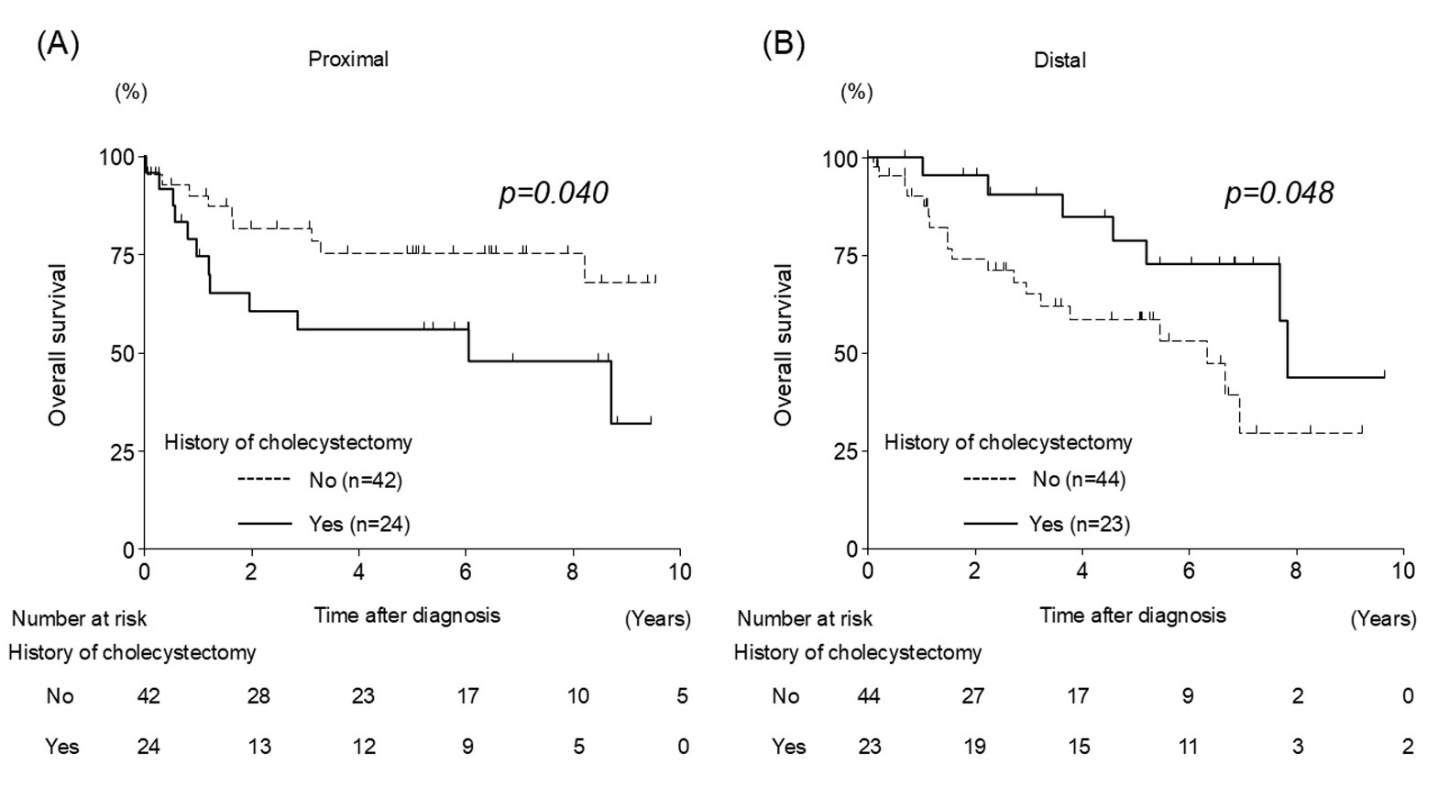
**

Kaplan-Meier curves of overall survival according to tumor location after propensity score matching including status of RAS mutation. (A) In proximal colorectal cancer, patients with a history of cholecystectomy had worse prognosis compared with those without (*p*=0.04). By contrast, in the distal colorectal cancer, patients with a history of cholecystectomy showed better overall survival (*p*=0.048).

**Supplementary figure 2**


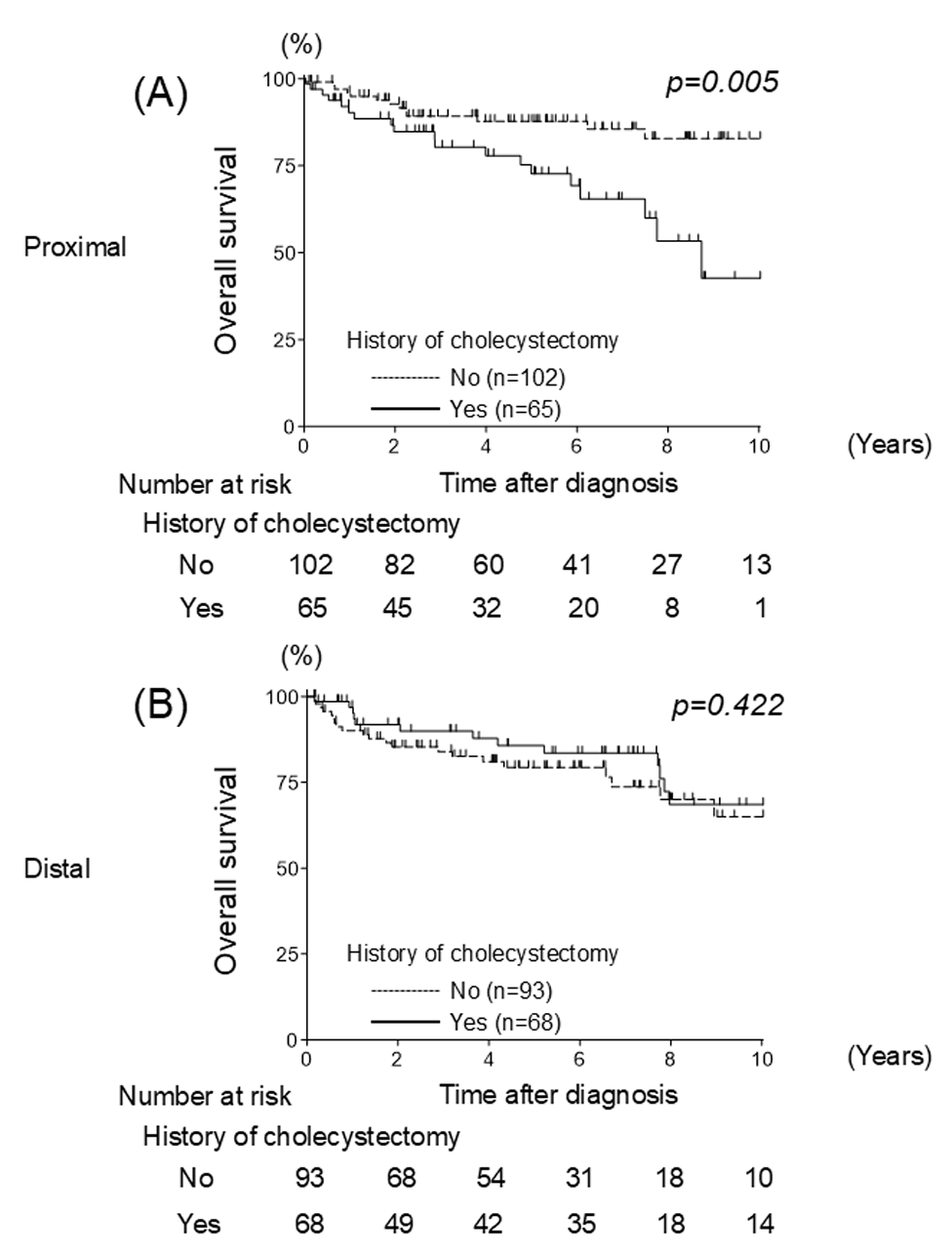


Kaplan–Meier curves of overall survival in patients who underwent resection according to the history of cholecystectomy. In resected cases, a history of cholecystectomy was associated with poorer survival in proximal CRC (*p*=0.005), whereas no such association was observed in distal CRC (*p*=0.422).

**Supplementary table 1.** Univariate and multivariate analyses of clinicopathologic variables in relation to overall survival after diagnosis in patients with distal colon cancer (n=2,112)

| Variables |  | Univariate analysis | |  | Multivariate analysis | |  |
| --- | --- | --- | --- | --- | --- | --- | --- |
|  | N | HR (95% CI) | *p*-value |  | HR (95% CI) | *p*-value* | |
| Sex, female | 781 | 0.89 (0.75–1.07) | 0.220 |  | - | - | |
| Age, ≥65 years | 1,424 | 1.72 (1.42–2.10) | <0.001 |  | 1.55 (1.20–1.99) | 0.001 | |
| Diabetes mellitus, yes | 386 | 1.30 (1.06–1.59) | 0.011 |  |  |  | |
| Clinical TNM stage, III–IV | 949 | 3.50 (2.91–4.23) | <0.001 |  | 1.86 (1.34–2.59) | <0.001 | |
| Poorly differentiation, yes | 60 | 3.45 (2.44–4.89) | <0.001 |  | 2.39 (1.48–3.88) | <0.001 | |
| Any RAS mutation, yes | 355 | 1.27 (1.01–1.61) | 0.041 |  | 1.37 (1.09–1.74) | 0.008 | |
| Initial treatment strategy, resection | 1,865 | 0.09 (0.07–0.11) | <0.001 |  | 0.15 (0.11–0.2) | <0.001 | |
| Cholecystectomy history, yes | 76 | 0.80 (0.50–1.26) | 0.337 |  | - | - | |

*The multivariate Cox regression model initially included sex (female vs. male), age (≥65 vs. <65 years), diabetes mellitus (yes vs. no), clinical TNM stage (III–IV vs. 0–II), tumor differentiation (poor vs. well/moderate), RAS mutation (yes vs. no), initial treatment strategy (resection vs. non-resection), cholecystectomy history (yes vs. no). Backward elimination was conducted with a threshold p of 0.05 to select variables for the final models.

Abbreviations: CI, confidence interval; HR, hazard ratio; TNM, tumor node metastasis.

**Supplementary table 2**. Comparison of the clinicopathological variables according to cholecystectomy history in patients with proximal colon cancer

|  | Before PSM | | |  | After PSM* | | |
| --- | --- | --- | --- | --- | --- | --- | --- |
| Variables | History of cholecystectomy | |  |  | History of cholecystectomy | |  |
|  | Yes (n=82) | No (n=1,293) | *p*-value |  | Yes (n=24) | No (n=42) | *p*-value |
| Sex, female | 36 (44%) | 46 (56%) | 0.420 |  | 13 (54%) | 26 (62%) | 0.539 |
| Age at diagnosis, years | 78 (72–84) | 74 (66–81) | 0.001 |  | 73 (69–77) | 75 (69–78) | 0.580 |
| Body mass index, kg/m^2^ | 20 (22–25) | 20 (22–25) | 0.977 |  | 23 (21–25) | 23 (20–25) | 0.743 |
| Diabetes mellitus, yes | 22 (27%) | 241 (19%) | 0.067 |  | 6 (25%) | 9 (21%) | 0.739 |
| Clinical TNM stage |  |  | 0.433 |  |  |  | 0.172 |
| 0 | 20 (24%) | 347 (27%) |  |  | 2 (8.3%) | 1 (2.4%) |  |
| I | 12 (15%) | 258 (20%) |  |  | 5 (21%) | 10 (24%) |  |
| II | 7 (9%) | 126 (9.9%) |  |  | 2 (8.3%) | 8 (19%) |  |
| III | 23 (28%) | 323 (25%) |  |  | 5 (21%) | 15 (36%) |  |
| IV | 20 (24%) | 224 (18%) |  |  | 10 (42%) | 8 (19%) |  |
| Tumor differentiation |  |  | 0.055 |  |  |  | 0.964 |
| Well | 39 (48%) | 690 (53%) |  |  | 10 (42%) | 20 (48%) |  |
| Moderate | 15 (18%) | 300 (23%) |  |  | 5 (21%) | 7 (17%) |  |
| Poorly | 11 (13%) | 82 (6.3%) |  |  | 6 (25%) | 10 (24%) |  |
| Undifferentiated | 17 (21%) | 221 (17%) |  |  | 3 (13%) | 5 (12%) |  |
| KRAS mutation, yes | 14 (58%) | 188 (45%) | 0.191 |  | 14 (58%) | 26 (63%) | 0.684 |
| NRAS mutation, yes | 0 (0%) | 9 (3%) | 0.454 |  | 0 (0%) | 1 (3.1%) | 0.449 |
| BRAF mutation, yes | 1 (10%) | 30 (17%) | 0.583 |  | 1 (11%) | 0 (0%) | 0.174 |
| MSI, high | 2 (22%) | 43 (23%) | 0.984 |  | 1 (17%) | 1 (11%) | 0.756 |
| Initial treatment strategy |  |  | 0.002 |  |  |  | 0.481 |
| Resection | 65 (79%) | 1,178 (95%) |  |  | 18 (75%) | 35 (83%) |  |
| Chemotherapy | 6 (11%) | 47 (3.6%) |  |  | 4 (17%) | 3 (7.1%) |  |
| Palliative care | 11 (14%) | 68 (5.3%) |  |  | 2 (9.1%) | 4 (9.5%) |  |

* Patients with and without history of cholecystectomy were matched in a 1:2 ratio by propensity score matching using the baseline characteristics (age, sex, history of diabetes mellitus, clinical TNM stage, tumor differentiation, RAS mutation status, and initial treatment strategy).

Abbreviations: MSI, microsatellite instability; PSM, propensity score matching; TNM, tumor node metastasis.

**Supplementary table 3.** Comparison of the clinicopathological variables according to cholecystectomy history in patients with distal colon cancer

|  | Before PSM | | |  | After PSM* | | |
| --- | --- | --- | --- | --- | --- | --- | --- |
| Variables | History of cholecystectomy | |  |  | History of cholecystectomy | |  |
|  | Yes (n=76) | No (n=2,036) | *p*-value |  | Yes (n=23) | No (n=44) | *p*-value |
| Sex, female | 28 (37%) | 753 (37%) | 0.980 |  | 5 (22%) | 7 (16%) | 0.555 |
| Age at diagnosis, years | 74 (68–80) | 70 (62–77) | <0.001 |  | 74 (64–79) | 72 (68–77) | 0.672 |
| Body mass index, kg/m^2^ | 23 (21–26) | 23 (20–25) | 0.204 |  | 24 (22–27) | 24 (21–26) | 0.951 |
| Diabetes mellitus, yes | 19 (25%) | 367 (18%) | 0.122 |  | 7 (30%) | 16 (36%) | 0.627 |
| Clinical TNM stage |  |  | 0.616 |  |  |  | 0.267 |
| 0 | 23 (30%) | 476 (23%) |  |  | 0 (8.3%) | 2 (4.5%) |  |
| I | 16 (21%) | 394 (19%) |  |  | 5 (22%) | 3 (6.8%) |  |
| II | 6 (7.9%) | 157 (7.7%) |  |  | 3 (13%) | 3 (6.8%) |  |
| III | 1 (1.3%) | 38 (1.9%) |  |  | 7 (30%) | 19 (43%) |  |
| IV | 0 (0%) | 16 (0.8%) |  |  | 8 (35%) | 17 (39%) |  |
| Tumor differentiation |  |  | 0.023 |  |  |  | 0.616 |
| Well | 48 (63%) | 1,185 (58%) |  |  | 8 (35%) | 22 (50%) |  |
| Moderate | 14 (18%) | 540 (27%) |  |  | 10 (44%) | 14 (32%) |  |
| Poorly | 6 (7.9%) | 54 (2.7%) |  |  | 3 (13%) | 6 (14%) |  |
| Undifferentiated | 8 (11%) | 257 (13%) |  |  | 2 (8.7%) | 2 (4.6%) |  |
| KRAS mutation, yes | 6 (25%) | 321 (40%) | 0.131 |  | 6 (26%) | 9 (21%) | 0.599 |
| NRAS mutation, yes | 0 (0%) | 29 (4.8%) | 0.398 |  | 0 (0%) | 0 (0%) | - |
| BRAF mutation, yes | 1 (9.1%) | 7 (1.9%) | 0.107 |  | 1 (9.1%) | 0 (0%) | 0.219 |
| MSI, high | 0 (0%) | 7 (1.9%) | 0.690 |  | 0 (0%) | 0 (0%) | - |
| Initial treatment strategy |  |  | 0.783 |  |  |  | 0.631 |
| Resection | 69 (91%) | 1,796 (88%) |  |  | 21 (91%) | 37 (84%) |  |
| Chemotherapy | 4 (5.3%) | 144 (7.1%) |  |  | 2 (8.7%) | 6 (7.1%) |  |
| Palliative care | 3 (4%) | 96 (4.7%) |  |  | 0 (0%) | 1 (2.3%) |  |

* Patients with and without history of cholecystectomy were matched in a 1:2 ratio by propensity score matching using the baseline characteristics (age, sex, history of diabetes mellitus, clinical TNM stage, tumor differentiation, RAS mutation status, and initial treatment strategy).

Abbreviations: MSI, microsatellite instability; PSM, propensity score matching; TNM, tumor node metastasis.

**Supplementary table 4**. Comparison of the clinicopathological variables according to cholecystectomy history in patients with proximal colon cancer who underwent resection

|  | Before PSM | | |  | After PSM* | | |
| --- | --- | --- | --- | --- | --- | --- | --- |
| Variables | History of cholecystectomy | |  |  | History of cholecystectomy | |  |
|  | Yes (n=65) | No (n=1,178) | *p*-value |  | Yes (n=65) | No (n=102) | *p*-value |
| Sex, female | 27 (42%) | 563 (48%) | 0.326 |  | 27 (42%) | 49 (48%) | 0.411 |
| Age at diagnosis, years | 77 (72–83) | 74 (66–81) | 0.003 |  | 77 (72–83) | 73 (69–80) | 0.580 |
| Body mass index, kg/m^2^ | 23 (21–25) | 23 (20–25) | 0.977 |  | 23 (21–25) | 23 (20–25) | 0.743 |
| Diabetes mellitus, yes | 20 (31%) | 230 (20%) | 0.028 |  | 20 (31%) | 36 (35%) | 0.546 |
| Clinical TNM stage |  |  | 0.915 |  |  |  | 0.924 |
| 0 | 20 (31%) | 347 (30%) |  |  | 20 (31%) | 29 (28%) |  |
| I | 12 (18%) | 257 (22%) |  |  | 12 (18%) | 19 (19%) |  |
| II | 7 (11%) | 117 (10%) |  |  | 7 (11%) | 9 (9%) |  |
| III | 20 (6%) | 317 (27%) |  |  | 20 (31%) | 31 (30%) |  |
| IV | 6 (9%) | 133 (11%) |  |  | 6 (9%) | 14 (14%) |  |
| Tumor differentiation |  |  | 0.041 |  |  |  | 0.924 |
| Well | 36 (55%) | 665 (56%) |  |  | 20 (42%) | 20 (48%) |  |
| Moderate | 10 (15%) | 270 (23%) |  |  | 12 (21%) | 7 (17%) |  |
| Poorly | 9 (14%) | 67 (6%) |  |  | 7 (25%) | 10 (24%) |  |
| Undifferentiated | 10 (15%) | 176 (15%) |  |  | (13%) | 5 (12%) |  |
| KRAS mutation, yes | 10 (56%) | 167 (45%) | 0.359 |  | 10 (56%) | 14 (41%) | 0.322 |
| NRAS mutation, yes | 0 (0%) | 8 (3%) | 0.530 |  | 0 (0%) | 1 (5%) | 0.435 |
| BRAF mutation, yes | 1 (10%) | 30 (17%) | 0.583 |  | 1 (14%) | 0 (0%) | 0.179 |
| MSI, high | 1 (14%) | 6 (86%) | 0.901 |  | 2 (29%) | 4 (37%) | 0.732 |

* Patients with and without history of cholecystectomy were matched in a 1:2 ratio by propensity score matching using the baseline characteristics (age, sex, history of diabetes mellitus, clinical TNM stage, tumor differentiation, RAS mutation status, and initial treatment strategy).

Abbreviations: MSI, microsatellite instability; PSM, propensity score matching; TNM, tumor node metastasis.

**Supplementary table 5.** Comparison of the clinicopathological variables according to cholecystectomy history in patients with distal colon cancer who underwent resection

|  | Before PSM | | |  | After PSM* | | |
| --- | --- | --- | --- | --- | --- | --- | --- |
| Variables | History of cholecystectomy | |  |  | History of cholecystectomy | |  |
|  | Yes (n=69) | No (n=1,796) | *p*-value |  | Yes (n=68) | No (n=93) | *p*-value |
| Sex, female | 25 (36%) | 666 (37%) | 0.886 |  | 25 (37%) | 33 (35%) | 0.867 |
| Age at diagnosis, years | 73 (68–80) | 70 (62–77) | 0.002 |  | 73 (68–80) | 71 (64–77) | 0.170 |
| Body mass index, kg/m^2^ | 23 (21–26) | 20 (23–25) | 0.282 |  | 23 (21–26) | 23 (20–25) | 0.897 |
| Diabetes mellitus, yes | 16 (23%) | 317 (18%) | 0.238 |  | 16 (24%) | 27 (29%) | 0.436 |
| Clinical TNM stage |  |  | 0.602 |  |  |  | 0.746 |
| 0 | 23 (34%) | 476 (27%) |  |  | 23 (25%) | 23 (34%) |  |
| I | 16 (24%) | 392 (22%) |  |  | 16 (24%) | 22 (24%) |  |
| II | 6 (9%) | 197 (11%) |  |  | 6 (12%) | 11 (9%) |  |
| III | 14 (21%) | 488 (28%) |  |  | 14 (26%) | 24 (21%) |  |
| IV | 9 (13%) | 219 (12%) |  |  | 9 (14%) | 13 (13%) |  |
| Tumor differentiation |  |  | 0.014 |  |  |  | 0.605 |
| Well | 45 (65%) | 1,091 (61%) |  |  | 45 (66%) | 53 (57%) |  |
| Moderate | 13 (19%) | 468 (26%) |  |  | 12 (18%) | 22 (24%) |  |
| Poorly | 5 (7%) | 35 (2%) |  |  | 5 (7%) | 6 (6%) |  |
| Undifferentiated | 6 (9%) | 202 (11%) |  |  | 6 (9%) | 12 (13%) |  |
| KRAS mutation, yes | 5 (23%) | 262 (39%) | 0.114 |  | 5 (24%) | 20 (45%) | 0.093 |
| NRAS mutation, yes | 0 (0%) | 25 (5%) | 0.393 |  | 0 (0%) | 1 (4%) | 0.466 |
| BRAF mutation, yes | 1 (9%) | 6 (2%) | 0.117 |  | 1 (9%) | 0 (0%) | 0.306 |
| MSI, high | 0 (0%) | 6 (2%) | 0.715 |  | 0 (0%) | 0 (0%) | - |

* Patients with and without history of cholecystectomy were matched in a 1:2 ratio by propensity score matching using the baseline characteristics (age, sex, history of diabetes mellitus, clinical TNM stage, tumor differentiation, RAS mutation status, and initial treatment strategy).

Abbreviations: MSI, microsatellite instability; PSM, propensity score matching; TNM, tumor node metastasis.
